# Pretreatment Serum Cystatin C Levels Predict Renal Function, but Not Tumor Characteristics, in Patients with Prostate Neoplasia

**DOI:** 10.1155/2017/7450459

**Published:** 2017-07-24

**Authors:** Feilong Yang, Dawei Li, Yu Di, Yongzhen Zhang, Yuanwei Zang, Juchao Ren, Lei Yan, Zunlin Zhou, Hainan Liu, Zhonghua Xu

**Affiliations:** Department of Urology, Qilu Hospital of Shandong University, Jinan 250012, China

## Abstract

To evaluate the role of Cystatin C (Cys-C) in tumorigenesis and progression of prostate cancer (PCa), we retrospectively collected the clinical information from the records of 492 benign prostatic hyperplasia (BPH), 48 prostatic intraepithelial neoplasia (PIN), and 173 PCa patients, whose disease was newly diagnosed and histologically confirmed. Pretreatment serum Cys-C levels were compared across the various groups and then analyzed to identify relationships, if any, with clinical and pathological characteristics of the PCa patient group. There were no significant differences in serum Cys-C levels among the three groups (*P* > 0.05). In PCa patients with normal SCr levels, patient age was correlated with serum Cys-C level (*P* ≤ 0.001) but did not correlate with alkaline phosphatase (AKP), lactate dehydrogenase (LDH), prostate specific antigen (PSA), Gleason score, or bone metastasis status (*P* > 0.05). Age and SCr contributed in part to the variations in serum Cys-C levels of PCa patients (*r *= 0.356,* P* ≤ 0.001;* r* = 0.520,* P* ≤ 0.001). In conclusion, serum Cys-C levels predict renal function in patients with prostate neoplasia, but were not a biomarker for the development of prostate neoplasia, and were not correlated with the clinicopathological characteristics of PCa.

## 1. Introduction

Prostate cancer (PCa) develops in the unique gland of the male reproductive system, where it becomes a detriment to men's health. In 2015, PCa was ranked the second most frequently diagnosed cancer in males worldwide and the fifth leading cause of cancer deaths in the world [1]. In the United States, it was estimated that 241,740 new cases developed in 2012, making it the most frequently diagnosed cancer type therein [2]. In addition, 28,170 deaths were attributed to PCa, accounting for more than ten percent of cancer deaths in men [2]. The incidence of PCa varies widely worldwide. PCa is least common in South and East Asia and most common in United States, with moderate incidences in Europe. Moreover, in China, it was estimated that the incidence of prostate cancer was ranked sixth and the mortality of prostate cancer was ranked seventh in men [3]. Though widely studied, the precise mechanism of prostate cancer has not yet been fully clarified and further investigation is needed.

Cystatin C (Cys-C), encoded by the CST3 gene, belongs to the type two cystatin superfamily and has been extensively studied since it was first described in 1961 [4, 5]. CST3 is located on the short arm of chromosome 20, spans 7.3 kb [6], contains four exons, encodes a 120-amino acid active cysteine proteinase inhibitor [7], and shares several features with housekeeping genes [6]. Cys-C is ubiquitously expressed in nucleated cells [8, 9] in tissues such as the testis, epididymis, seminal vesicle, and prostate [10] and is then secreted into various human fluids to inhibit the activity of cysteine proteases such as papain and cathepsins B, H, K, and L [11]. Moreover, Cys-C is considered to function as a p53-inducible tumor suppressor and apoptotic mediator that negatively regulates cathepsin L activity during carcinogenesis [9]. Therefore, Cys-C is believed to play a critical role in the tumor suppressive function of p53 [9], as well as in extracellular, protein homeostasis. An imbalance between Cys-C and cysteine proteinases has been observed in the pathogenesis of a broad spectrum of diseases [12, 13], including cancer [14–17]. However, the diagnostic role of Cys-C in cancers, such as renal cell carcinoma [18] and pancreatic tumors [18], has been dismissed. Recent studies by Wegiel et al. and Jiborn et al. indicated that Cys-C was downregulated in PCa specimens [19, 20]. Cys-C was also found to modulate the invasion of PCa cells by means of the androgen receptor and MAPK/Erk2 pathways [19]. Aberrant expression of Cys-C is associated with neuroendocrine differentiation in PCa [20]. Studies from another group also revealed that serum Cys-C levels may distinguish PCa patients from BPH patients and functioned as an indicator for the treatment of metastatic PCa with zoledronic acid in a small patient group [15, 21]. Taken together, published studies have reported both positive [15, 21, 22] and negative [23] effects of serum Cys-C levels on predicting malignancies. Thus, the feasibility of using serum Cys-C levels in cancer detection remains controversial.

We evaluated the diagnostic significance of circulating Cys-C levels in patients with prostate neoplasia, including BPH, PIN, and PCa. We also explored the relationship between serum Cys-C levels and clinicopathological characteristics of PCa patients.

## 2. Materials and Methods

### 2.1. Patient Population

The study was reviewed and approved by the Ethics Committee at Qilu Hospital of Shandong University and the Approval Number is KYLL-2015(KS)-156. We retrospectively collected clinical and pathological information from the records of inpatients that were newly diagnosed with prostate neoplasia and treated at the Department of Urology, Qilu Hospital of Shandong University between February 2010 and September 2013. Histologic confirmation of BPH, PIN, or PCa was obtained for all patients. None of the patients received preoperative hormonal therapy or radiotherapy. Patients with clinical characteristics such as (a) coexistence of prostate neoplasia and malignancy of other tissues or organs, (b) histologically diagnosed PCa but not adenocarcinoma, or (c) inadequate clinical information were excluded.

### 2.2. Sample Test and Data Collection

After an overnight fast, 5 mL of venous blood was obtained from patients with prostate neoplasia and assayed immediately before clinical treatment. Blood samples were deposited into test tubes containing a clot activator and gel, allowed to clot at room temperature, and subsequently centrifuged at 2000 rpm for 10 min. Serum was then collected to determine the concentration of Cys-C and other biochemical markers. The circulating Cys-C levels were tested with immunoturbidimetric method using a Roche Cobas 8000 analyzer with reagents purchased from BioSino Bio-Technology & Science Inc., Beijing, China. The levels of SCr, AKP, and LDH were determined using a Roche Cobas 8000 analyzer with reagents purchased from Roche. PSA was quantified using a Roche Cobas 601 analyzer, also with Roche reagents. The tests were completed according to the manufacturers' instructions. Clinical data, including age, SCr, Cys-C, PSA, AKP, LDH, Gleason score, and ECT (Bone Imaging), were retrieved from patient files. We first compared pretreatment serum Cys-C levels among all patients in the three groups. Subsequently, subclass analyses were conducted to exclude the possible effects of renal function on the pretreatment levels of Cys-C. Furthermore, we performed statistical analyses to compare the BPH and PIN groups. We also analyzed the association of serum Cys-C levels with clinical characteristics of PCa patients. Linear correlations among age, SCr, and Cys-C were also evaluated.

### 2.3. Statistical Analysis

The normal distribution of quantitative data in the various groups was assessed by the Kolmogorov-Smirnov test. Normally distributed data were expressed as the mean ± standard deviation (SD), while the median (range) was reported for data not following a Gaussian distribution. Statistical analyses were accordingly performed using the parametric Student's *t*-test, one-way ANOVA, or the nonparametric Mann–Whitney *U* test and Kruskal-Wallis *H* test. Qualitative data were reported as numbers and percentages, and the Pearson *χ*^2^ test was used to compare differences among various groups. Pearson correlation coefficients were calculated to examine the associations among age, SCr, and Cys-C. Data were analyzed and processed using the Statistical Package for Social Sciences version 16.0 (SPSS 16.0, SPSS Inc., Chicago, IL, USA). All probabilities (*P*) were two-tailed and *P* values less than 0.05 were considered statistically significant.

## 3. Results

### 3.1. Characteristics of Study Population

The study group consisted of patients with prostate neoplasia consecutively presenting at the Department of Urology, Qilu Hospital of Shandong University. In total, 492 BPH, 48 PIN, and 173 PCa patients conforming to inclusive criteria were eligible for inclusion in the final study. The ages of eligible BPH, PIN, and PCa patients were 70.50 ± 7.55, 70.35 ± 7.95, and 70.88 ± 8.01 years, respectively (*P* = 0.775). All patients were further grouped according to their levels of SCr (*P* = 0.916). Clinical characteristics of enrolled patients are presented in Table 1.

### 3.2. Serum Cys-C Levels in Patients with Prostate Neoplasia

**Figure 1:**
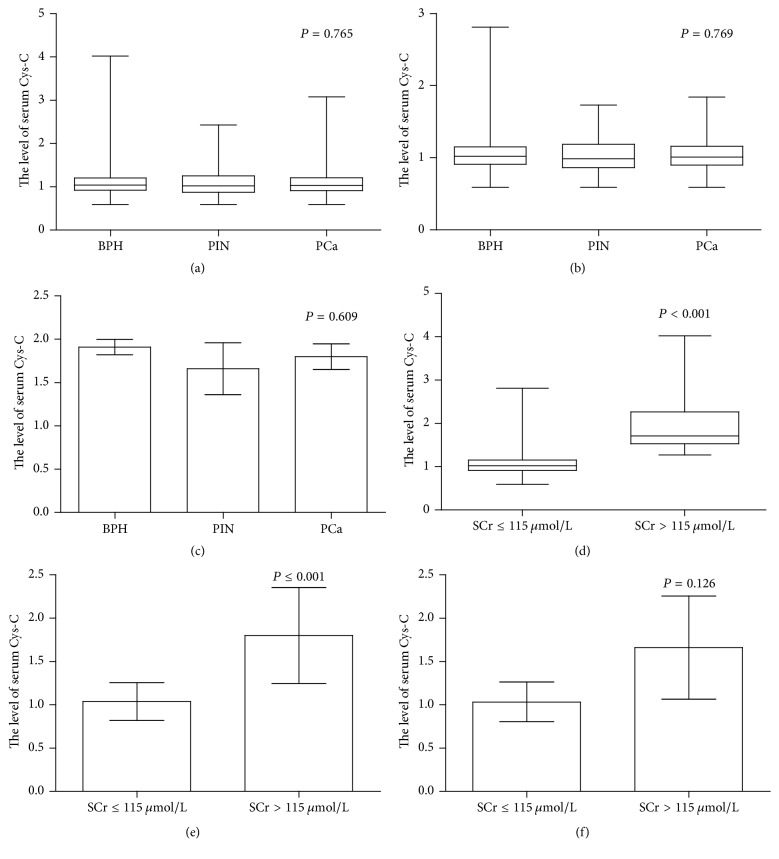
The comparisons of serum Cys-C in various groups of patients with prostate neoplasia. The comparison of serum Cys-C in total patients with prostate neoplasia (*P* = 0.765) (a); in patients with normal SCr (*P* = 0.769) (b); in patients with high SCr (*P* = 0.609) (c); in BPH patients (*P* < 0.01) (d); in PCa patients (*P* ≤ 0.001) (e); in PIN patients (*P* = 0.126) (f). *P*: parametric Student's *t*-test.

The levels of serum Cys-C were 1.04 (0.59–4.02), 1.02 (0.59–2.43), and 1.03 (0.59–3.08) mg/L in the BPH, PIN, and PCa groups, respectively (*P* = 0.765) (Figure 1(a)). Patients were then further grouped and analyzed according to their levels of SCr (less or greater than 115 *μ*mol/L) (*P* = 0.916) (Table 1). There were insignificant associations with serum Cys-C levels among patients with SCr levels less than 115 *μ*mol/L (*P* = 0.769) (Figure 1(b)) or greater than 115 *μ*mol/L (*P* = 0.609) (Figure 1(c)). Levels of serum Cys-C were higher in BPH (*P* < 0.001) and PCa (*P* ≤ 0.001) patients with SCr levels greater than 115 *μ*mol/L (Figures 1(d) and 1(e)) than those in the PIN group. Moreover, the levels of serum Cys-C in PIN patients with SCr levels greater than 115 *μ*mol/L were similar to those in patients with SCr levels less than 115 *μ*mol/L (*P* = 0.126) (Figure 1(f)).

### 3.3. Association of Serum Cys-C Levels with Clinical Characteristics of PCa

**Figure 2:**
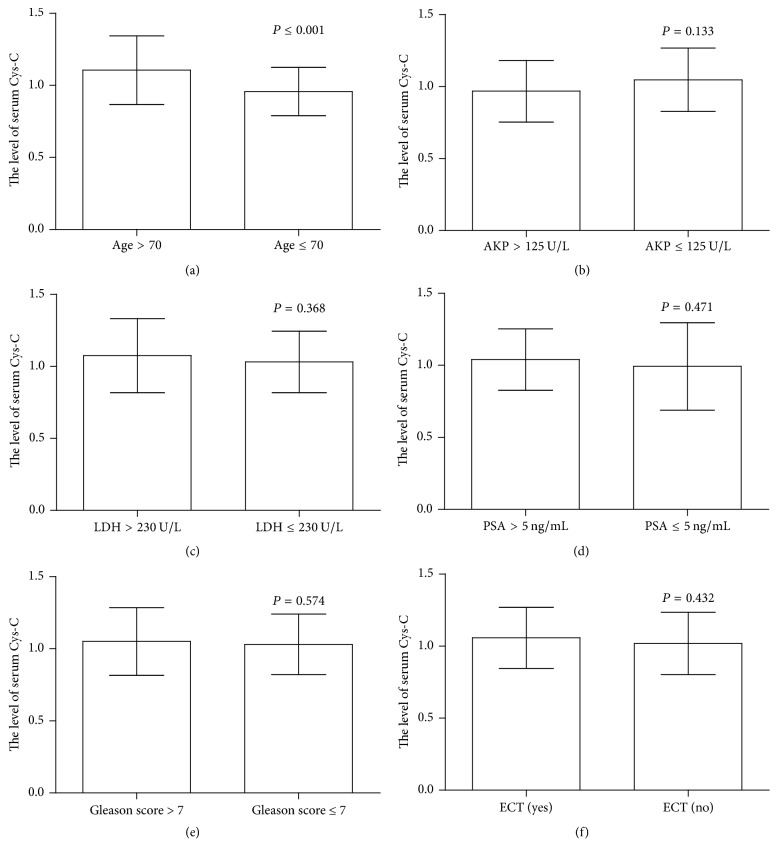
The comparisons of serum Cys-C in subgroup analyses of PCa patients with normal SCr. The subgroup comparison of serum Cys-C in patients with normal SCr based on patient age (*P* ≤ 0.001) (a); AKP (*P* = 0.133) (b); LDH (*P* = 0.368) (c); PSA (*P* = 0.471) (d); Gleason score (*P* = 0.574) (e); the status of bone metastasis (*P* = 0.432) (f). *P*: parametric Student's *t*-test.

Considering the effect of SCr on levels of serum Cys-C, the associations between serum Cys-C levels and clinical characteristics of 159 PCa patients with normal SCr (less than 115 *μ*mol/L) were further evaluated. PCa patients were stratified accordingly, and these data are presented in Table 2. We found that the levels of serum Cys-C in older PCa patients (more than 70 years) were higher than in younger patients (*P* ≤ 0.001) (Figure 2(a)). Moreover, there were insignificant associations between the levels of serum Cys-C and clinical characteristics, such as AKP, LDH, PSA, Gleason score, and bone metastasis status (all *P* > 0.05) (Figures 2(b)–2(f)).

### 3.4. Levels of Serum Cys-C Correlate with SCr and Age in PCa Patients

**Figure 3:**
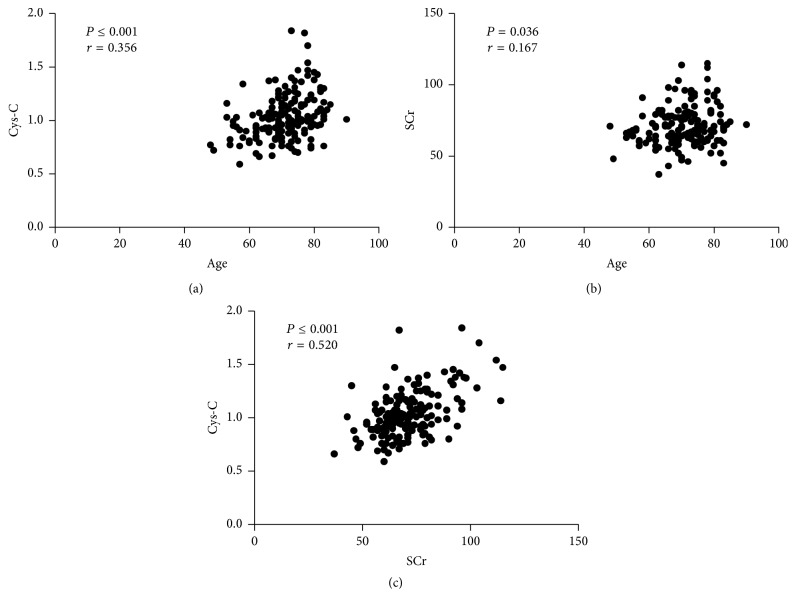
Correlations of serum Cys-C, SCr, and age in patients with PCa (normal SCr). Correlation of serum Cys-C and patient age (*r* = 0.356, *P* ≤ 0.001) (a); correlation of SCr and patient age (*r* = 0.167, *P* = 0.036) (b); correlation of SCr and serum Cys-C (*r* = 0.520, *P* ≤ 0.001) (c). *P*: statistical significance; *r*: correlation coefficient according to Pearson correlation test.

Using linear correlation analyses, there were positive correlations between circulating Cys-C levels, age (*r* = 0.356, *P* ≤ 0.001) (Figure 3(a)), and SCr (*r* = 0.167, *P* = 0.036) (Figure 3(b)) in PCa patients. In addition, the SCr levels of PCa patients correlated with their pretreatment levels of serum Cys-C (*r* = 0.520, *P* ≤ 0.001) (Figure 3(c)).

## 4. Discussion

Cys-C is a cationic, nonglycosylated protein with a molecular mass of 13 kDa. It is ubiquitously expressed in all nucleated cells [8], widely distributed in human biological fluids [9], freely filtered through renal glomeruli, and uniquely and almost completely reabsorbed and catabolized in the proximal tubules [18]. Therefore, its classic role as a sensitive marker for renal function has been extensively studied [24–27] and further confirmed in a meta-analysis [28]. In addition to its role in predicting kidney function, Cys-C is also a marker for inflammation [12], infection [13], tumorigenesis [16], prostate cancer pathological grade [20], malignant progression [14, 17], and several other processes [29, 30]. In the present study, we collected and analyzed clinical information to evaluate the diagnostic significance of circulating Cys-C in patients with prostate neoplasia and explored the relationship between serum Cys-C levels and clinicopathological characteristics of PCa patients. To our knowledge, this study is one of the first studies to focus on alterations circulating Cys-C concentrations in patients with PIN.

In the present study, there were no significant differences in the levels of serum Cys-C among all patients in the three groups (*P* = 0.765). Our result was in accordance with that from another study focusing on ovarian cancer, which excluded the role of serum Cys-C level as possible biomarker [23]. To exclude the impact of renal function on Cys-C levels, all patients were further grouped based on their SCr levels. Again, no differences in the levels of serum Cys-C were detected among the three prostate neoplasia groups in either the high SCr (*P* = 0.609) or normal SCr (*P* = 0.769) groups. However, a recent study found that the level of serum Cys-C could distinguish PCa from BPH patients [15]. The conflicting results between these two studies may be attributed to differences in the ages of the patient groups. In our study, patient age was normally distributed, and there were no significant differences among the three groups (*P* = 0.775). However, in the study by Tumminello et al. [15], PCa patients (72.4 ± 7.8 years) were much older than BPH patients (62.8 ± 6.2 years). Age-related reductions in the glomerular filtration rate (GFR) [31, 32] lead to age-dependent increases in the concentrations of serum Cys-C [16]. Still, SCr affected the levels of serum Cys-C in both the BPH (*P* = 0.001) and PCa (*P* ≤ 0.001) groups. However, SCr did not affect the level of serum Cys-C in the PIN group (*P* = 0.126). These results could be explained by the small number of PIN patients with SCr levels greater than 115 *μ*mol/L (*n* = 4). However, the mean serum Cys-C level in PIN patients with high SCr (1.66 ± 0.60 *μ*mol/L) was higher than that in PIN patients with normal SCr levels (1.03 ± 0.23 *μ*mol/L).

We next investigated the possible relationship between circulating Cys-C and clinicopathological parameters in PCa patients with normal SCr. Unfortunately, when PCa patients were stratified according to levels of AKP, LDH, PSA, bone metastasis status, and Gleason score, no significant differences in serum Cys-C levels were found among the various groups (all *P* > 0.05). Our results were consistent with those of a previous study containing relatively few subjects [15]. However, we found that PCa patients older than seventy years of age had higher serum Cys-C levels than their younger counterparts (1.10 ± 0.24 versus 0.96 ± 0.17 mg/L, *P* ≤ 0.001). As discussed above, older age may alter renal function as well as levels of serum Cys-C [33]. Next, the possible relationships among serum Cys-C, age, and SCr were tested using linear correlation analyses. As expected, serum Cys-C levels were positively correlated with patient age (*r* = 0.356, *P* ≤ 0.001) and SCr (*r* = 0.520, *P* ≤ 0.001). Moreover, we found that patient age was positively correlated with SCr (*r* = 0.167, *P* = 0.036).

Taken together, the value of serum Cys-C levels as a feasible predictor for PIN and PCa was limited for the following reasons. First, Cys-C is a housekeeping protein ubiquitously expressed in all nucleated cells and highly expressed in the male reproductive system. Unlike PSA, its expression was actually downregulated in prostate tumors and its circulating level may be affected by complex mechanisms. Second, prostate neoplasia was common in older males. Age-dependent reductions in GFR and declines in renal function would confound with changes in the levels of serum Cys-C [16]. Third, the male urethra traverses through the center of the prostate gland. Obstruction of the urethra caused by a prostate lesion may impair kidney function, which may impact levels of circulating Cys-C.

In conclusion, no statistically significant differences in the levels of serum Cys-C were found among the BPH, PIN, and PCa groups. Circulating Cys-C was not a potential marker for prostate tumorigenesis and was not a reliable predictor for clinicopathological characteristics of PCa patients. The increases in serum Cys-C levels in the elderly PCa group may be partly ascribed to age-dependent reductions in GFR.

## Figures and Tables

**Table 1:** Clinical characteristics of patients with prostate neoplasia.

Characteristics	BPH	PIN	PCa	*P* value
Patients (*n*)	492	48	173	
Age (y)	70.50 ± 7.55	70.35 ± 7.95	70.88 ± 8.01	0.775^a^
Cys-C (mg/L)	1.04 (0.59–4.02)	1.02 (0.59–2.43)	1.03 (0.59–3.08)	0.765^b^
SCr (*n*, %)				
≤115 *μ*mol/L	455(92.5)	44(91.7)	159(91.9)	0.916^c^
>115 *μ*mol/L	37(7.5)	4(8.3)	14(8.1)	

BPH: benign prostatic hyperplasia; PIN: prostate intraepithelial neoplasia; PCa: prostate cancer; Cys-C: cystatin C; SCr: serum creatinine; *P*^a^: one-way ANOVA test; *P*^b^: Kruskal-Wallis *H* test; *P*^c^: Pearson *χ*^2^ test.

**Table 2:** The level of serum Cys-C in PCa patients with normal SCr.

Characteristics	*n* (%)	Mean ± SD	*P* value
Patients (n)	159 (100)	1.04 ± 0.22	
Age			0.000^a*∗*^
≤70 y	73 (45.91)	0.96 ± 0.17	
>70 y	86 (54.09)	1.10 ± 0.24	
AKP			0.133^*∗*^
>125 U/L	20 (12.58)	0.97 ± 0.21	
≤125 U/L	139 (87.42)	1.05 ± 0.22	
LDH			0.368^*∗*^
>230 U/L	24 (15.09)	1.07 ± 0.26	
≤230 U/L	135 (84.91)	1.03 ± 0.21	
PSA			0.471^*∗*^
>5 ng/mL	147 (92.45)	1.04 ± 0.21	
≤5 ng/mL	12 (7.55)	0.99 ± 0.30	
Gleason score			0.574^b*∗*^
≤5	4 (2.52)	1.03 ± 0.21	
6	22 (13.84)		
7	65 (40.88)		
8	40 (25.16)	1.05 ± 0.23	
9	21 (13.21)		
10	1 (0.63)		
Missing information	6 (3.77)	—	
ECT (bone metastasis)			0.432^c*∗*^
Yes	27 (16.98)	1.06 ± 0.21	
No	55 (34.59)	1.02 ± 0.12	
Possible	26 (16.35)	—	
Unknown	51 (32.08)	—	

SCr: serum creatinine; PCa: prostate cancer; Cys-C: cystatin C; *P*^a^ < 0.05; *P*^b^: Gleason score ≤ 7 versus Gleason score > 7; *P*^c^: bone metastasis versus non-bone metastasis; *P*^*∗*^: Student's *t*-test.
